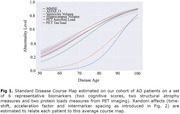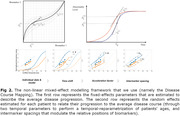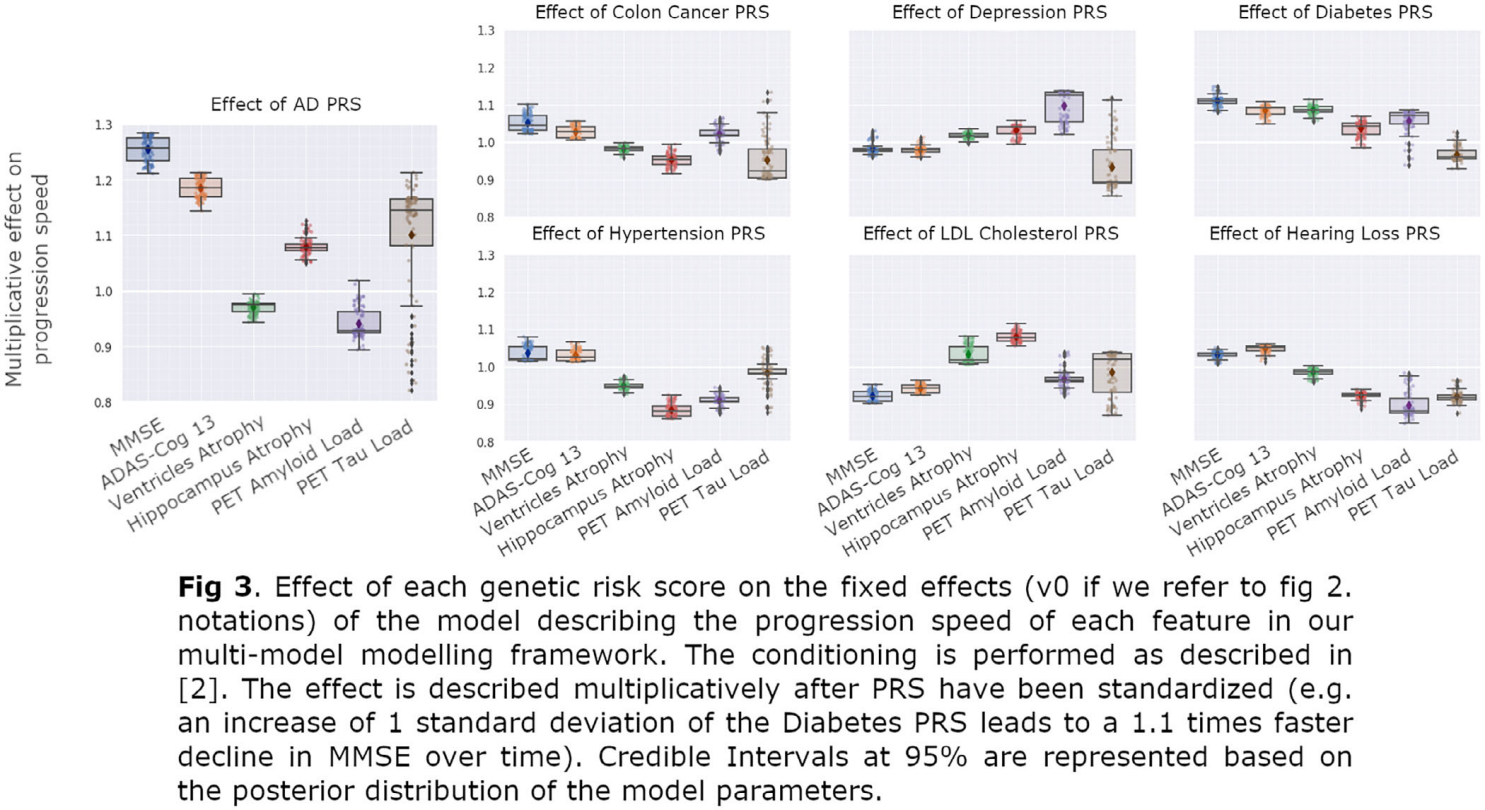# Genetic Risks of AD and Associated Health Conditions are Linked with the Dynamic Patterns of AD’s Clinical and Physiological Course

**DOI:** 10.1002/alz.090441

**Published:** 2025-01-03

**Authors:** Nemo Fournier, Stanley Durrleman

**Affiliations:** ^1^ Paris Brain Institute, PARIS France

## Abstract

**Background:**

Over‐representation of several health conditions (such as diabetes, hearing loss, etc) have been identified up to 15 years before Alzheimer’s Disease (AD) diagnosis through the study of electronic health records [1]. Mechanisms underlying these associations remain elusive. We propose to study the associations between these co‐pathologies (proxied by genetic risk scores), and the physiological and clinical evolution of AD patients.

**Method:**

Longitudinal and genetic data was obtained on 1120 AD patients from the ADNI cohort. Genetic risks for AD and selected health conditions (type 2 diabetes, hypertension, hearing loss, depression, ldl cholesterol, and colon cancer) are computed using state‐of‐the‐art Polygenic Risk Scores. We utilize a non‐linear mixed‐effects model [2] to estimate a multimodal progression profile of the disease (jointly modelling structural and PET imaging biomarkers as well as cognitive scores — *fig. 1*). Interpretable parameters describing each patient’s progression are estimated (*Fig. 2*). Pearson correlations between random‐effects and genetic risk scores were computed, with presented P‐values Bonferroni‐corrected for multiple testing. Conditioning the fixed‐effects of the model by the genetic risk scores (as introduced in [2]) allows studying associations between these genetic risks and the disease dynamics at a biomarker‐by‐biomarker level.

**Result:**

Higher AD genetic risk is associated with all longitudinal aspects of the disease: earlier onset (P<0.001), faster disease pace (P<0.001), and inter‐marker patterns (P = 0.042). Elevated type‐II diabetes genetic risk is associated with earlier onset of AD (p = 0.031). Increased hypertension genetic risk is associated with a faster progression pace (P = 0.012). We then exhibit (*Fig. 3*) finer links between these genetic risks and the progression patterns of the disease.

**Conclusion:**

This study unveils the relationship between genetic risk scores for health conditions and nuanced aspects of AD progression. We show that despite being established on case/control cohorts, these genetic risk scores offer valuable insights into the finer dynamics of disease progression.

**References**:

[1] Nedelec et al., *Identifying health conditions associated with Alzheimer’s disease up to 15 years before diagnosis: an agnostic study of French and British health records*, in Lancet Digital Health, 2022

[2] Fournier and Durrleman, *A Multimodal Disease Progression Model for Genetic Associations with Disease Dynamics*, in MICCAI 2023